# Supplementary material for: Theoretical study on the toxicity of ‘Novichok’ agent candidates
Source: R Soc Open Sci. 2019 Aug 7;6(8):190414. doi: 10.1098/rsos.190414 (PMC6731729; doi:10.1098/rsos.190414)
Supplement: Electronic Supplementary Material [file rsos190414supp1.pdf]

Electronic Supplementary Material

Theoretical study on the toxicity of “Novichok” agent candidates

Keunhong Jeong[a], Junwon Choi[b]

[a]Department of Chemistry and Nuclear & WMD Protection Research Center, Korea Military Academy, Seoul 01805, South Korea

[b] Chemical Kinomics Research Center, Korea Institute of Science and Technology, Seoul 02792, South Korea

Table of contents

P=O bond distance of each Novichok agent S3

P-F bond distance of each Novichok agent S3

The structure of A234 proposed by Mirzayanov S4

Comparison of Frequencies (cm–1) Related to P Atom in each calculated level of theory including experimental data. S5

Calculated scaring factor and R-squared value by comparison of calculated IR spectra and experimental IR spectra at each level of theory S6

Geometrical parameters of studied structures S7-S22

Novichok agent candidate	P=O (Å)		
	$B3LYP_1$	$B3LYP_2$	$M06\text{-}2X_2$
**A230**	1.48771	1.46336	1.45565
**A232**	1.48782	1.46381	1.45604
**A234**	1.48812	1.46366	1.45610

B3LYP/6-311++G(d,p) denoted as $B3LYP_1$ denotes, B3LYP/6-311++G(2d,2p) denoted as $B3LYP_2$, and M06-2X/6-311++G(2d,2p) denoted as $M06\text{-}2X_2$ denotes.

Novichok agent candidate	P-F (Å)		
	$B3LYP_1$	$B3LYP_2$	$M06\text{-}2X_2$
**A230**	1.57856	1.56019	1.54448
**A232**	1.57961	1.56158	1.54519
**A234**	1.58079	1.56171	1.54562

B3LYP/6-311++G(d,p) denoted as $B3LYP_1$ denotes, B3LYP/6-311++G(2d,2p) denoted as $B3LYP_2$, and M06-2X/6-311++G(2d,2p) denoted as $M06\text{-}2X_2$ denotes.

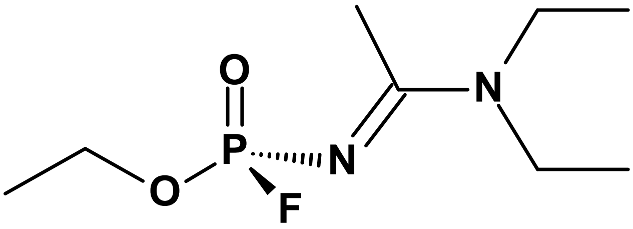

Chemical Structure of A-234 proposed by Mirzayanov

	Experimental data			
	P=O	P–C	P–O	P–F
GA	1286		792	
GB	1303	775	1016	840
GD	1279	844	988	754
VX	1227	729	964	

	B3LYP/6-311++G(d,p)			
	P=O	P–C	P–O	P–F
GA	1270		785	
GB	1279	753	992	799
GD	1282	797	986	747
VX	1218	750	925	

	B3LYP/6-311++G(2d,2p)			
	P=O	P–C	P–O	P–F
GA	1282		792	
GB	1293	757	995	807
GD	1291	805	989	748
VX	1230	723	994	

	M06-2X/6-311++G(2d,2p)			
	P=O	P–C	P–O	P–F
GA	1317		832	
GB	1324	792	1046	844
GD	1325	814	1044	841
VX	1274	778	1029	

Comparison of Frequencies (cm^{-1}) Related to P Atom in each calculated level of theory including experimental data.

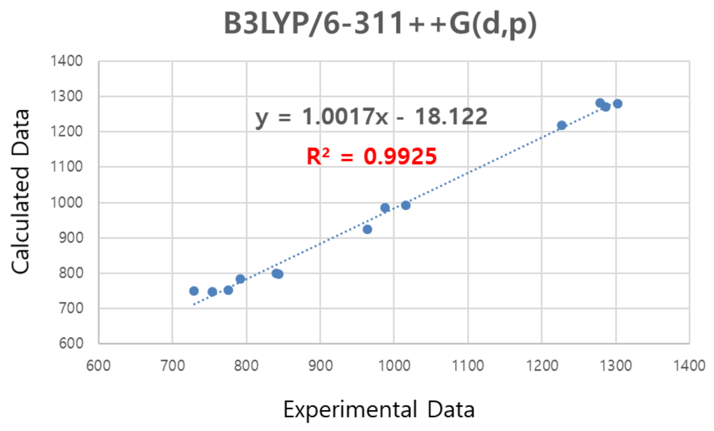

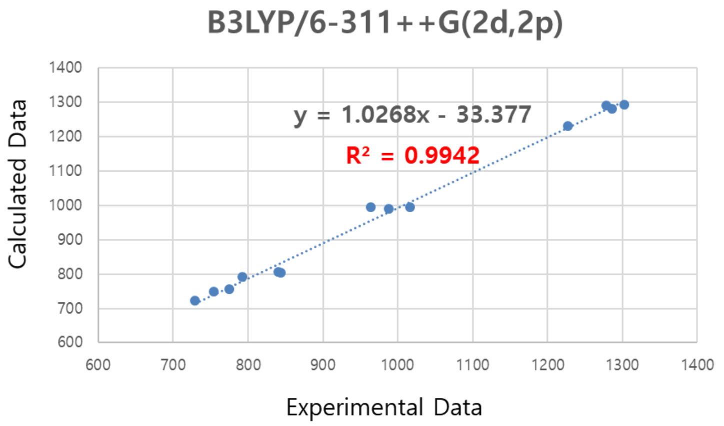

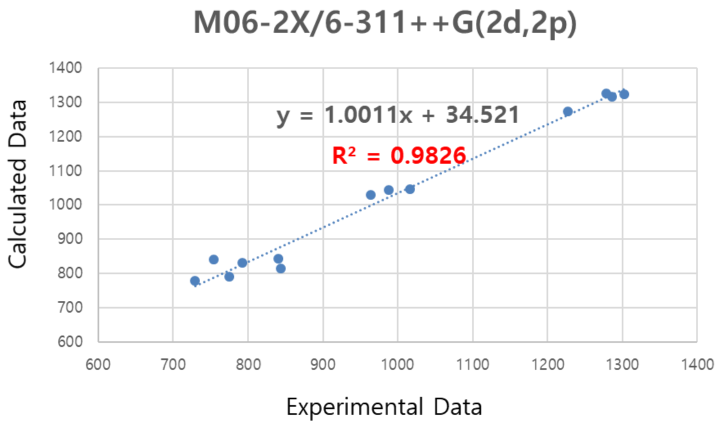

Calculated scaring factor and R-squared value by comparison of calculated IR spectra and experimental IR spectra at each level of theory

Coordinates of GA

P	-0.82166673	0.44589106	-0.24287997
O	-1.59143378	1.04689625	0.88183974
O	-0.13150184	-0.96376760	-0.01438259
C	-0.89119751	-2.18240599	0.33443918
H	-1.56042962	-1.93870503	1.16021656
C	0.10419142	-3.25041572	0.71261734
H	0.77817021	-3.47452200	-0.11716130
H	0.69359948	-2.94959096	1.58158964
H	-1.47730463	-2.46459061	-0.54209453
H	-0.44227813	-4.16253500	0.96778178
C	-1.99556717	0.10771257	-1.59580304
N	-2.72248038	-0.03312339	-2.48463291
N	0.39545642	1.36674042	-0.86118652
C	1.18867795	0.83834390	-1.99032606
H	0.65598963	0.95549509	-2.94039918
H	2.12245901	1.39967944	-2.03996191
H	1.42377201	-0.21183981	-1.83217832
C	0.14224782	2.81908579	-0.96371241

H	-0.41417511	3.06563900	-1.87425749
H	-0.41054717	3.16970816	-0.09437776
H	1.10543682	3.33031718	-0.99151623

Coordinates of GB

P	-1.14726689	0.12293238	0.17158117
O	-1.51191772	0.25833044	1.61055255
F	0.43926942	0.40186174	0.01141917
O	-1.33108805	-1.31251135	-0.47741985
C	-1.08045913	-2.59621105	0.24116526
H	-0.82006770	-2.34658750	1.27090047
C	0.08180475	-3.27942384	-0.45078575
H	0.30109612	-4.22315540	0.05568053
H	-0.16204656	-3.49647865	-1.49425279
H	0.97879344	-2.65723496	-0.41827648
C	-2.37659374	-3.38000345	0.20373088
H	-3.17967445	-2.83004319	0.69885028
H	-2.67246344	-3.59061784	-0.82744176
H	-2.23949834	-4.33146177	0.72444126

C	-1.87763482	1.25463511	-0.99619487
H	-2.95896861	1.10778351	-0.99355889
H	-1.47943018	1.06838402	-1.99428827
H	-1.64660783	2.27516069	-0.68659916

Coordinates of GD

P	-1.29051396	-0.05175260	0.33432467
O	-2.14172081	0.04521799	1.55440945
F	0.26284758	-0.10624803	0.78735625
O	-1.46224997	-1.35570407	-0.55154016
C	-1.76884508	-2.71518569	-0.00661429
H	-2.09015095	-2.57008104	1.02478376
C	-0.48408359	-3.55201453	-0.07878172
H	-0.45766114	-4.06905727	-1.04437838
H	0.36571089	-2.86429293	-0.06582381
C	-2.92040676	-3.22787377	-0.84914869
H	-3.79531148	-2.58231209	-0.74539579
H	-2.63179278	-3.26903532	-1.90293040
H	-3.19657569	-4.23520763	-0.53249190

C	-1.31803389	1.30285520	-0.82334551
H	-0.62665838	1.10678117	-1.64362483
H	-1.02652199	2.21546133	-0.30057219
H	-2.33235563	1.41256205	-1.21052224
C	-0.25201203	-4.58604008	1.05670873
C	-1.41278639	-5.59014718	1.17349654
H	-1.17810376	-6.35089816	1.92483509
H	-2.34400472	-5.10413799	1.47805858
H	-1.59122242	-6.10339672	0.22337372
C	1.03685136	-5.35545918	0.71165672
H	1.27402450	-6.08302424	1.49408713
H	0.93005672	-5.89900524	-0.23263341
H	1.88916136	-4.67538713	0.61361264
C	-0.05511701	-3.87015967	2.40571663
H	0.17941376	-4.59607493	3.19067306
H	0.76953710	-3.15212051	2.35365247
H	-0.95262299	-3.32930253	2.71922490

Coordinates of VX

P	-2.53286002	-0.35825811	0.33416839
O	-2.98829232	0.62660855	-0.87387146
S	-1.21098599	-1.68245463	-0.64768420
C	-3.95561002	-1.38452612	0.75069271
O	-1.95175422	0.39241024	1.50518549
C	-3.70829089	1.87464234	-0.59229408
C	0.26113820	-0.57833810	-0.91414165
C	-3.97501913	2.55819390	-1.91252988
C	1.25356999	-0.62078757	0.25279946
N	2.43215065	0.20214352	-0.02442031
C	3.46472173	-0.50147657	-0.82256475
C	2.90216796	1.02546563	1.11617361
C	4.33599555	0.47956827	-1.61454927
C	4.34814232	-1.49881314	-0.05008159
C	3.05393510	0.29030025	2.46371184
C	2.00130137	2.25547826	1.29472211
H	-3.66802773	-2.10612742	1.51702675
H	-4.73811214	-0.73371601	1.14844127
H	-4.32840565	-1.90431082	-0.13311647

H	-3.08620613	2.48971160	0.05989227
H	-4.63777226	1.62775712	-0.07356645
H	-4.50902501	3.49478548	-1.72915118
H	-3.04013101	2.79221004	-2.42765651
H	-4.59243638	1.93331577	-2.56233415
H	0.70344416	-0.95081804	-1.83857222
H	-0.09980799	0.43364536	-1.09743123
H	1.51405723	-1.66734225	0.46588362
H	0.74854933	-0.24042363	1.13944346
H	2.89992738	-1.08161139	-1.55883997
H	3.89185106	1.38952138	0.83162331
H	5.00603646	-0.07301008	-2.27993038
H	3.72473860	1.14743845	-2.22723913
H	4.96197043	1.09308112	-0.96037581
H	3.75151125	-2.19253432	0.54791656
H	4.93700465	-2.09195621	-0.75674760
H	5.04831892	-0.98430619	0.61359921
H	3.51452190	0.96151190	3.19534012
H	2.08458513	-0.01425792	2.86944780

H	3.68270303	-0.59695855	2.38348295
H	2.37413130	2.88686342	2.10725421
H	1.96818557	2.85836313	0.38361726
H	0.97600834	1.96644836	1.54732203

Coordinates of A230

P	-1.63966716	1.96713463	2.08010990
O	-2.72122227	2.92075039	2.37818603
F	-1.06819295	1.24851286	3.36418491
O	-1.92094079	0.80492668	1.06042954
C	-3.27239456	0.30211935	0.76069835
H	-3.74219090	-0.01875773	1.68922157
H	-3.84043163	1.10751011	0.29843083
C	-3.05426403	-0.85586678	-0.18840160
H	-2.49185873	-1.65975209	0.27924216
H	-2.57416462	-0.53538427	-1.10943303
Cl	-4.67667305	-1.53724858	-0.64441321
O	-0.20520321	2.56420757	1.59555714
N	-0.31371025	3.31092267	0.39882748

C	0.80949560	3.80178875	0.06893468
Cl	2.31223814	3.66941968	0.86530414
F	0.84528330	4.52135741	-1.03490100

Coordinates of A232

P	-1.66817126	0.34690426	-0.52278559
O	-1.83150354	-1.02042806	0.00032699
F	-2.18813178	0.51161096	-2.00524267
O	-2.30189114	1.56154383	0.23718353
C	-3.33381326	1.58560406	1.31637144
H	-3.51006814	2.65523940	1.41437776
C	-4.58687264	0.90906182	0.77231566
H	-4.48988746	-0.17395586	0.73769532
H	-4.84156401	1.30339229	-0.20835616
Cl	-6.00573320	1.26974122	1.85161180
O	-0.14792767	0.84021748	-0.85548839
N	0.68261296	0.80340720	0.28670830
C	1.87029984	1.14353953	-0.00403096
Cl	2.51286584	1.59861369	-1.51747702

F	2.74893606	1.15220465	0.97920221
C	-2.77020940	1.00804293	2.59514980
H	-2.58154646	-0.06331284	2.50329467
H	-1.84291191	1.51899180	2.85956372
H	-3.48493772	1.16582802	3.40556565

Coordinates of A234

P	-2.54443866	1.28369673	-0.39751322
O	-3.15302115	0.00565664	-0.80483851
F	-3.48091864	2.52575028	-0.67892328
O	-2.03650593	1.49292919	1.06866985
C	-2.41194152	0.80242104	2.34132085
H	-1.67300143	1.21512958	3.02702001
C	-3.80821127	1.30428322	2.73224461
H	-4.55124594	0.92181898	2.03212926
Cl	-4.24993225	0.50617634	4.33691556
O	-1.30560796	1.83773295	-1.30538845
N	-0.21792102	0.93685527	-1.33363180
C	0.71854880	1.37924748	-2.06730361

Cl	0.78932615	2.83834412	-2.94914607
F	1.79908913	0.63115555	-2.17616960
C	-2.24061430	-0.69737187	2.20777547
H	-3.03780796	-1.14997755	1.61668970
H	-1.27703086	-0.91592856	1.74401998
H	-2.24232914	-1.14815752	3.20045773
C	-3.91085789	2.80858708	2.89730362
H	-3.19133495	3.17288967	3.63410727
H	-3.71066198	3.30130428	1.94359098
H	-4.91651808	3.08846308	3.21411951

Coordinates of HF

F	-0.07789602	0.45166403	0.00000000
H	-1.00812607	0.45166403	0.00000000

Coordinates of Serine

N	-2.50046308	-2.55087763	-0.48516740
H	-1.71954531	-2.81107713	0.11359802
C	-3.05127095	-1.27016330	-0.03247731

H	-3.92467078	-1.02946352	-0.63860360
C	-3.46549753	-1.29352908	1.44529634
C	-2.00530239	-0.19084903	-0.24748450
H	-4.20446527	-2.09084227	1.57654306
H	-2.59071842	-1.51764632	2.06600013
O	-4.02469418	-0.02269301	1.80338845
O	-0.83824454	-0.30352899	0.07383409
H	-4.26053435	-0.05263261	2.73878589
H	-3.21116521	-3.26824721	-0.36534946
O	-2.50066483	0.91909631	-0.81814573
H	-1.79243966	1.58310417	-0.89252113

Coordinates of A230-Serine Complex

P	-1.52927189	1.62153497	2.34384219
O	-2.58532694	2.59509239	2.71159476
O	-1.95395470	0.52531077	1.27140405
C	-3.15499693	0.62476517	0.44242805
H	-4.00531445	0.88150778	1.07254505
H	-2.99803686	1.39378277	-0.31291237

C	-3.31770953	-0.74550085	-0.18147853
H	-3.48259074	-1.51309008	0.57035350
H	-2.47275002	-1.00408678	-0.81462818
Cl	-4.79055971	-0.72625533	-1.25097910
O	-0.13687640	2.28116950	1.75405234
N	-0.35887457	3.01681052	0.57621564
C	0.70807115	3.55784572	0.15736745
Cl	2.27833863	3.49294192	0.82941308
F	0.62873638	4.27976317	-0.94611055
O	-0.80675630	0.81793693	3.49922837
C	-1.53373518	0.29921284	4.66096473
C	-0.50590585	-0.25216361	5.65065087
H	-2.19703359	-0.50223049	4.33393496
H	-2.10817575	1.11020210	5.11071744
N	-1.14261978	-0.77691230	6.85999297
H	0.05801786	-1.05104210	5.17138874
C	0.45723344	0.85628223	6.05071755
H	-1.72564724	-0.05425498	7.27708984
H	-1.75910889	-1.54255188	6.60006042

O	0.14776671	1.79434222	6.75754558
O	1.68484825	0.69679665	5.53610357
H	2.24612626	1.44391453	5.81042975

Coordinates of A232-Serine Complex

P	-1.82546637	0.08408976	-0.44745688
O	-1.98715535	-1.13397558	0.38293405
O	-2.16940074	1.49061517	0.18700217
C	-3.21210214	1.85224006	1.17804211
H	-3.10740640	2.93528587	1.21886508
C	-4.56782800	1.49809239	0.57654835
H	-4.75989938	0.42663512	0.59378336
H	-4.65834068	1.88658711	-0.43420766
Cl	-5.90386531	2.27620962	1.53926337
O	-0.28630896	0.34079413	-0.98392480
N	0.67188539	0.31626195	0.04599989
C	1.84064815	0.51518792	-0.40323620
Cl	2.33791112	0.78958075	-2.01595642
F	2.83323838	0.51722806	0.46924941

C	-2.91512516	1.23701033	2.52989275
H	-3.05204186	0.15456666	2.51953387
H	-1.89136372	1.46896756	2.82882020
H	-3.58891318	1.66507761	3.27487981
O	-2.57462457	0.11069643	-1.84647110
C	-2.62686018	-1.05823153	-2.72714288
C	-3.26432602	-0.62843026	-4.05221979
H	-3.24403829	-1.82351314	-2.25646901
H	-1.61463499	-1.43397464	-2.88893917
N	-3.41051136	-1.75524734	-4.97409126
H	-4.24723840	-0.20506149	-3.84878246
C	-2.40296319	0.43829460	-4.71083870
H	-2.50382185	-2.19395005	-5.12070746
H	-4.01175128	-2.45366374	-4.54430639
O	-1.36535752	0.20323653	-5.29689069
O	-2.89876344	1.67561381	-4.56373800
H	-2.28823825	2.31399922	-4.97355149

Coordinates of A234-Serine Complex

P	-2.75803604	1.35656348	-0.46729392
O	-3.12171576	0.07881763	-1.12648970
O	-1.98831844	1.31157315	0.91156787
C	-2.37044026	0.69587174	2.20525211
H	-1.40294272	0.57943964	2.69317437
C	-3.19702921	1.74221050	2.96938758
H	-4.20272943	1.80245517	2.55618203
Cl	-3.45031819	1.08621491	4.67896212
O	-1.72300041	2.28942732	-1.35427215
N	-0.56980041	1.60157922	-1.77382085
C	0.24580929	2.37808367	-2.35630436
Cl	0.10056600	4.05439493	-2.66179248
F	1.37266917	1.85006768	-2.80187838
C	-3.03755634	-0.65215904	2.01377019
H	-4.03112289	-0.55288349	1.57014667
H	-2.42146996	-1.28968904	1.37820230
H	-3.14157263	-1.14387445	2.98141132
C	-2.55167201	3.11067999	3.06361292
H	-1.54848665	3.04655404	3.49181734

H	-2.47760014	3.54747067	2.06575608
H	-3.15901853	3.77561152	3.67944558
O	-3.92302168	2.41050953	-0.25001628
C	-5.01928873	2.60022689	-1.20217791
C	-5.36973961	4.09249132	-1.27536442
H	-5.87795366	2.04677344	-0.82325135
H	-4.73651587	2.22023330	-2.18489339
N	-6.53320485	4.32698762	-2.12938351
H	-5.58543078	4.45092982	-0.26902228
C	-4.18560447	4.87313314	-1.82283517
H	-6.33939644	3.98270087	-3.06727029
H	-7.31682349	3.78702535	-1.77131170
O	-3.93887121	4.99021596	-3.00644860
O	-3.42205394	5.40582490	-0.85799303
H	-2.65852917	5.85227587	-1.26516742
